# Superior Oblique Anterior Transposition with Horizontal Recti Recession-Resection for Total Third-Nerve Palsy

**DOI:** 10.1155/2015/780139

**Published:** 2015-11-11

**Authors:** Muhsin Eraslan, Eren Cerman, Sumru Onal, Mehdi Suha Ogut

**Affiliations:** ^1^Department of Ophthalmology, School of Medicine, Marmara University, 34890 Istanbul, Turkey; ^2^Department of Ophthalmology, School of Medicine, Koç University, Istanbul, Turkey; ^3^Department of Ophthalmology, V.K. Foundation, American Hospital, Istanbul, Turkey; ^4^Acıbadem Hospital, Istanbul, Turkey

## Abstract

*Aims*. To report the results of lateral rectus muscle recession, medial rectus muscle resection, and superior oblique muscle transposition in the restoration and maintenance of ocular alignment in primary position for patients with total third-nerve palsy. *Methods*. The medical records of patients who underwent surgery between March 2007 and September 2011 for total third-nerve palsy were reviewed. All patients underwent a preoperative assessment, including a detailed ophthalmologic examination. *Results*. A total of 6 patients (age range, 14–45 years) were included. The median preoperative horizontal deviation was 67.5 Prism Diopter (PD) (interquartile range [IQR] 57.5–70) and vertical deviation was 13.5 PD (IQR 10–20). The median postoperative horizontal residual exodeviation was 8.0 PD (IQR 1–16), and the vertical deviation was 0 PD (IQR 0–4). The median correction of hypotropia following superior oblique transposition was 13.5 ± 2.9 PD (range, 10–16). All cases were vertically aligned within 5 PD. Four of the six cases were aligned within 10 PD of the horizontal deviation. Adduction and head posture were improved in all patients. All patients gained new area of binocular single vision in the primary position after the operation. *Conclusion*. Lateral rectus recession, medial rectus resection, and superior oblique transposition may be used to achieve satisfactory cosmetic and functional results in total third-nerve palsy.

## 1. Introduction

Oculomotor nerve palsy, in contrast to paralysis of the fourth or sixth cranial nerves that may affect the function of only one extraocular muscle, may affect the function of four of the six extraocular muscles. In complete third-nerve palsy the eye is fixed in a position of abduction, depression, and intorsion, because of the two remaining functional muscles, the lateral rectus and the superior oblique. In partial third-nerve palsy any or all of these functions may be incompletely effected. Ptosis may occur due to paralysis of levator palpebrae.

The surgical correction of oculomotor nerve palsy remains a challenge for ophthalmologists. The main goal of surgery in total third-nerve palsy is to align the paralyzed eye in a primary position, regardless of which surgical procedure is employed. Restoring adduction and elevation functions is often secondary in importance. Various surgical techniques have been described to achieve this goal [1].

Examples include modified Jensen and modified Hummelsheim operations or horizontal rectus muscle surgeries and superior transposition of these muscles, with or without tenotomy of the superior oblique muscle. Other modalities used are globe fixation procedures which may include periosteal flap fixation of the globe [2] and the suture/T-plate anchoring platform systems [3].

However, transposition of the superior oblique muscle, along with horizontal recti surgery, is another technique which is used to address total third-nerve palsy [4–8].

We report herein the results of surgeries undertaken for the treatment of total third-nerve palsy.

## 2. Materials and Methods

This is an observational case series of patients who underwent surgery for total nerve palsy. We reviewed the medical records of six patients who were evaluated at our pediatric ophthalmology and strabismus clinic from March 2007 to September 2011. This study is conducted in accordance with the amended Declaration of Helsinki and ethical clearance was obtained from the Human Research Ethics Committee of our university. The written permissions and informed consent were obtained from the patients to publish the photographs. All six patients underwent lateral rectus muscle recession, combined with medial rectus muscle resection and superior oblique muscle transposition. Only patients with a minimum of six months' postoperative follow-up were included in the study. All the patients had functioning lateral rectus and superior oblique muscles. Demographic features, etiology of third-nerve palsy, and clinical features of the patients are shown in Table 1.

The preoperative assessment included a detailed ophthalmologic examination, including best corrected visual acuity, an external examination, a slit-lamp examination, tonometry, a fundus examination, and an assessment of ocular motility. Deviations were determined in prism diopters (PD) with a Krimsky test. The Worth four-dot test at 6 m was used to assess diplopia in the primary position. Surgery was performed at least after six months of onset.

One of the authors (MSO) performed all the surgeries under general anesthesia using an operation microscope. The final decision about the surgical dose was made peroperatively according to the forced-duction test. Using a limbal conjunctival approach, conventional lateral rectus recession was applied. The scleral fixation of the muscle was carried out with a double-armed 6.0 Vicryl suture (Vicryl, Ethicon, Somerville, NJ). Medial rectus muscle resection was then performed. Afterwards, the eye was deviated inferiorly, and the medial conjunctival incision was extended superiorly to isolate the superior oblique muscle tendon under the superior rectus. The tendons of the superior oblique muscle were isolated and cut at the medial margin of the superior rectus muscle. Then, if necessary, a resection was performed to shorten and tighten the muscle sufficiently to ensure the eye was in the primary position when it was fixed to the superior edge of the medial rectus insertion. The conjunctival incisions were closed in the conventional way. Postoperatively, commercially available topical corticosteroid-antibiotic drops were prescribed for two weeks.

The amount of recession and resection of the horizontal rectus muscles was recorded. Deviations were measured at the first day, one week, one month, and six months after the surgery, as well as during the patient's last follow-up visit. Ocular deviations were measured by placing prisms in front of the operated eye. They were determined in PD by an alternate cover test for far and near fixation. Primary outcome measures were those defined in previous studies: the achievement of horizontal alignment within 10 PD and vertical alignment within 5 PD of orthophoria in the primary position, without the head position and relief of diplopia [9, 10].

The percentage change in the angle of deviation following the surgery for far and near fixation was reported as a secondary outcome measure. It was calculated using the preoperative and postoperative deviation obtained at the last examination.

At least after six months following the strabismus surgery, when the alignment was stabilized, ptosis was repaired in required patients with prediction of probable postoperative diplopia due to ptosis correction.

The analysis was carried out with the Statistical Package for Social Sciences (SPSS) Windows version 17.0 (SPSS for Windows Inc., Chicago, IL, USA).

## 3. Report of Cases

### 3.1. Patient Number 3

**Figure 1:**
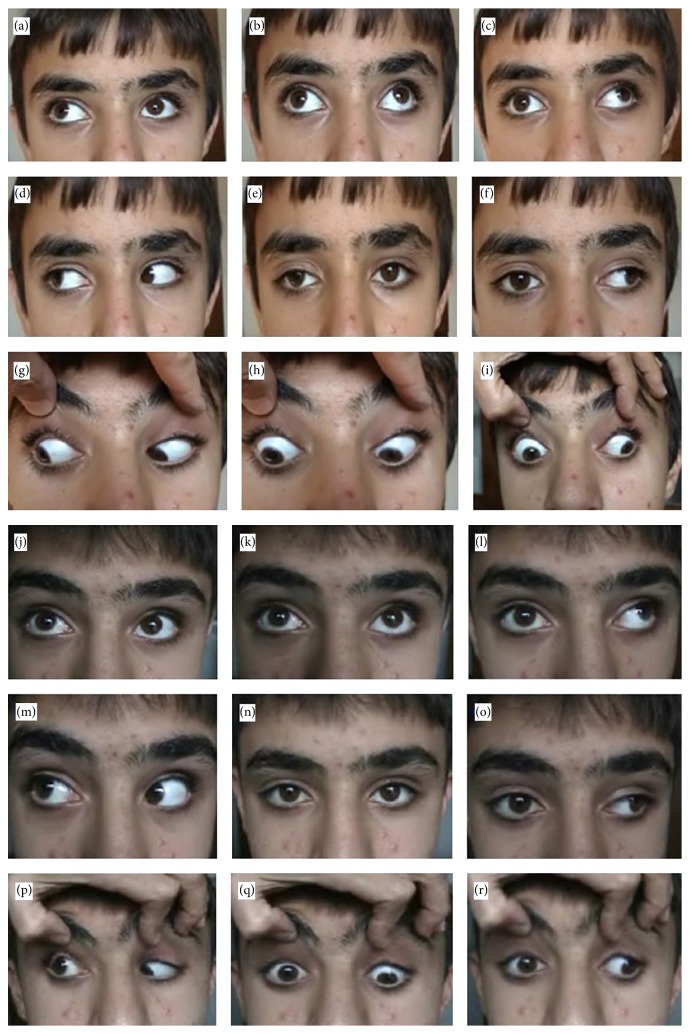
Preoperative (a–i) and postoperative (j–r) ocular alignment photographs of a right partial oculomotor nerve palsy in nine gaze positions (Tables 1 and 2, patient number 3). Limitation of adduction on left gaze (f), mild ptosis, and exotropia (e) is seen in preoperative pictures. Postoperative photographs showing both eyes are aligned in primary position (n), limited adduction function on left gaze (o), and right lateral gaze normal (m). A minimal limitation at inferior gaze occurred (q).

A 14-year-old male presented with congenital total third-nerve palsy in the right eye. He had no history of previous ocular surgeries. On examination, he had mild ptosis and anisocoria. His best corrected visual acuity was 0.6 in the right eye and 1.0 (Snellen chart) in the left eye. Preoperative examination of eye movements revealed limitations in adduction, elevation, and depression in the right eye, but ductions were normal in the left eye. In the primary gaze position, he had exotropia of 65 PD and hypotropia of 12 PD (Figure 1). No pathological changes were identified with cranial MRI. There was a marked secondary deviation in the contralateral eye when fixing with the right eye. All movements, except abduction, were grossly limited in the right eye. Ductions of the noninvolved eye were normal. A peroperative forced-duction test revealed no restriction. He underwent a 7 mm recession of the right lateral rectus muscle, a 5 mm resection of the right medial rectus, and superior oblique transposition to the superior edge of the medial rectus.

On the first postoperative day, the patient had 8 PD exotropia, and his eyes were vertically aligned. He had stable alignment within 10 PD of orthotropia in follow-up examinations at one week, one month, and six months (Figure 1).

### 3.2. Patient Number 6

**Figure 2:**
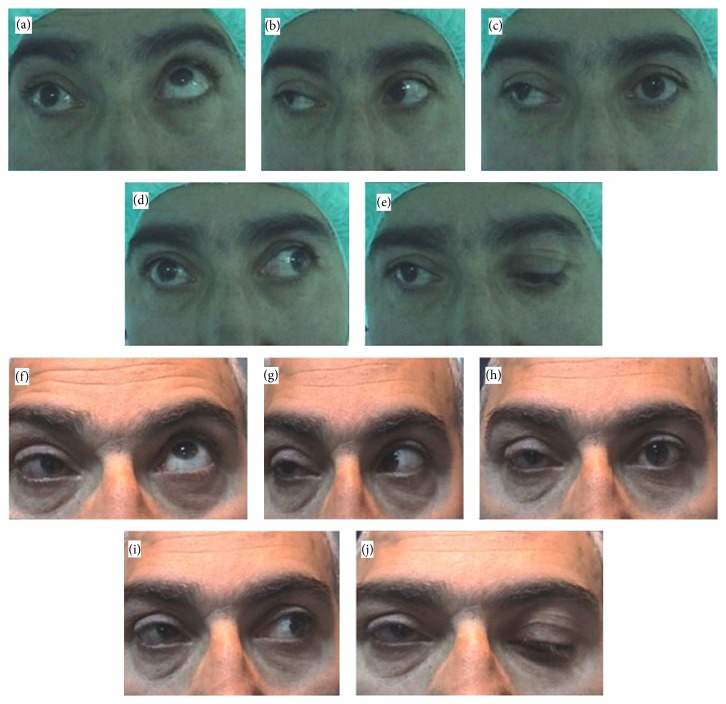
Preoperative (a–e) and postoperative (f–j) ocular alignment of right third-nerve palsy (patient number 6 in Tables 1 and 2). The patient is fixing with the left eye and mild ptosis is seen in photograph (c). A mild upper lid retraction with downgaze may be the evidence of aberrant regeneration (e). In the right eye all movements, except abduction, were grossly limited (a–e). No deviation is seen on postoperative photographs in primary position on sixth postoperative month (h). Postoperative superior and inferior gaze are grossly limited but there is a limited function at abduction (f-g–j) and a limited adduction function is regained (i).

A 45-year-old male presented with total third-nerve palsy in his right eye. He had a history of hypophyseal macroadenoma and a gamma-knife procedure. Postoperatively, ptosis and squint developed. He had no history of previous ocular surgeries. Best corrected visual acuity in the right and left eye was 0.8 and 1.0 (Snellen chart), respectively. He fixated with the left eye. Mild upper lid retraction on downgaze indicated possible aberrant regeneration. In the right eye, all movements, except abduction, were grossly limited. Ductions in the noninvolved eye were normal. There was no evidence of restriction in a forced-duction test. Preoperatively, he had exotropia of 60 PD and hypotropia of 10 PD (Figure 2). He underwent a 9 mm recession of the right lateral rectus muscle, a 6 mm right medial rectus resection, and superior oblique resection and transposition.

On the first postoperative day, the patient had orthophoria, and his eyes were horizontally and vertically aligned. The ptosis was not covering the optical axis and he had no diplopia in primary position. One week, one month, and six months after the surgery, he had orthophoria in a primary position (Figure 2).

## 4. Results

**Figure 3:**
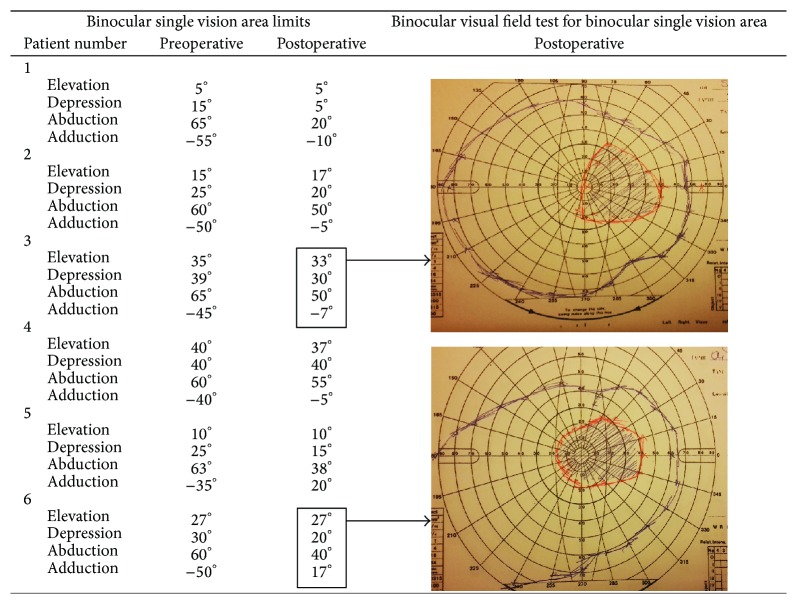
Preoperative, postoperative binocular single vision area limits and binocular visual field test for binocular single vision area of patients number 3 and number 6. Goldman kinetic visual field test results are shown with the black arrows for patients number 3 and number 6. All patients gained a new area of binocular single vision in the primary position after the operation. Also the binocular single vision areas are enlarged for all patients like what is shown for patients number 3 and number 6.

The median age of the patients was 36.5 years (age range, 14–45 years). The median follow-up time was 37 (6–54) months. The median preoperative horizontal exodeviation was 67.5 PD (interquartile range [IQR] 57.5–70) and vertical deviation was 13.5 PD (IQR 10–20), respectively. The median postoperative horizontal residual exodeviation was 8.0 PD (IQR 1–16), and the vertical deviation was 0 PD (IQR 0–4). The median correction of hypotropia was 13.5 ± 2.9 PD (range, 10–16) with superior oblique transposition. All the cases were aligned within 5 PD of vertical deviation, and four of the six cases were aligned within 10 PD of horizontal deviation. Adduction and head posture improved in all the patients. The horizontal diplopia was compensated by head deviation in patients with postoperative horizontal deviation over 10 PD (patients 1 and 2). The preoperative and postoperative patient data and the resection-recession amounts are summarized in Table 2. The preoperative and postoperative limitations of ocular movement are summarized in Table 3. Preoperative and postoperative binocular single vision area limits of all study patients and binocular visual field test results of patients number 3 and number 6 are shown in Figure 3.

## 5. Discussion

Oculomotor nerve palsy is a well-defined disorder. However, the best surgical treatment technique remains controversial. In the surgical treatment of third-nerve palsy, as in any paralytic strabismus, the surgeon has to balance the muscular forces acting on the globe [11]. In third-nerve palsies, like in all other cranial nerve palsies, if the palsy persists after the acute phase, surgery may be beneficial, if the patient understands the postoperative expectations well and agrees to undergo surgery. The aim of the surgery should be to eliminate the diplopia, to achieve a good cosmetic appearance in the primary position, and if possible to restore adduction function or to create or relocate a binocular single vision area to the most suitable place for the patient.

Many different types of surgical procedures have been described in the literature [1, 4–7, 12–19]. In the absence of medial rectus function, supramaximal resection of the medial rectus, combined with supramaximal recession of the lateral rectus, is mostly insufficient to align the primary gaze in third-nerve palsies. In total paralysis, adduction will not be gained without medial transposition of a functioning muscle. Therefore, most authors advocate transposition procedures [4, 13].

In a study conducted in 1996, Von Noorden [20] suggested transposition of the vertical recti to the insertion of the medial rectus following tenotomy of the lateral rectus and the superior oblique muscles. Peter [16] and later Aoki et al. [17] described a different technique involving fracturing the trochlea and attaching the shortened superior oblique tendon to the medial rectus insertion. Scott [7] modified this procedure by transposing the superior oblique tendon without trochleatomy. As shown in the study of Harley [6] more adduction is achieved using the procedure proposed by Jackson than that proposed by Scott. However, Maruo et al. [12] reported cosmetically satisfactory results with Scott's procedure when combined with recession of the lateral and superior recti. Gottlob et al. [4] achieved satisfactory long-term results with Scott's procedure, combined with recession of the lateral rectus, in seven patients with total third-nerve palsy. We are of the opinion that it is important to assess the functions of all muscles affected in partial third-nerve palsy before deciding the right trade-off for this procedure. Any transposition procedure would not create a new force; it only may change or possibly increase the binocular single vision area. As an example in patient 3 the procedure created a limitation in depression; however patient gained a new area of binocular single vision in the primary position as given in example in Figure 3.

Arblaster and Burke also reported that cosmetic as well as substantial subjective and objective functional improvements can be achieved in longstanding strabismus, even in cases of Chiari II malformation causing bilateral internuclear ophthalmoplegia and unilateral third-nerve palsy. They showed the enlarged and centralized binocular single vision area following surgeries using Goldman visual field testing similar to our report. Their patient reported significant functional improvements in daily activities even when minimal objective improvements were achieved in adduction and primary position alignment [21].

In the technique presented herein, vertical recti are preserved, and the procedure is applied only to the affected eye and completed in one session. We achieved cosmetically and functionally satisfactory outcomes in all patients. All cases were vertically aligned within 5 PD, and four cases were horizontally aligned within 10 PD. All patients were eventually satisfied with their cosmetic appearance. The patients also gained some adduction function. The horizontal diplopia was compensated by head deviation in two patients with postoperative horizontal deviation over 10 PD (patients 1 and 2). Two cases (patients 3 and 5) showed a limitation in the inferior gaze that could be compensated with a 10-degree head tilt.

The median correction of hypodeviation following superior oblique transposition was found to be 13.5 ± 2.9 (10–16) PD. We are of the opinion that superior oblique transposition adds about 15 to 20 PD (median 16 ± 4.7) corrections of exodeviation when compared with the standard surgical dose table [22]. Other types of procedures, including horizontal recti surgeries alone, can also help in achievement of orthophoria in primary position for short-term, but most cases show disruption of alignment in long-term. Modified Scott's procedure can help in achievement of orthophoria and most importantly helps in stabilization of alignment in primary position for long-term.

Although we obtained satisfactory results with the procedure described herein, it has some limitations, including postoperative hyperdeviation, which was within 5 PD. But patient satisfaction was generally good because all patients increased their binocular single vision area eventually. Hypertropia and extorsion may occur due to the loss of superior oblique muscle function. Hypertropia can be resolved secondarily with a superior rectus muscle recession, and hypotropia can be resolved with an inferior rectus recession [23]. More studies should be done to optimize the localization of the superior oblique muscle in transposition.

Blepharoptosis is a common finding in patients with third cranial nerve palsy [24]. Ptosis correction in such patients is challenging and most of the patients need multistep surgical plan. The possibility of postoperative diplopia following ptosis correction procedure must be kept in mind before deciding for surgery. The examination for diplopia has to be done with upper eyelids lifted to consider whether existing ptosis prevents the underlying diplopia. In this kind of patients with an underlying diplopia the correction may lead to disturbance of vision and may reduce patient's quality of life. Although the surgical treatment of ptosis due to third cranial nerve palsy is difficult, an appropriate approach and previously planned multiple interventions might achieve acceptable results in considerable number of patients [25]. In our case series we followed the patients at least six months after the strabismus surgery; ptosis was repaired in required patients after a detailed diplopia examination as described previously when the alignment was stabilized.

Not evaluating stereopsis and torsion is the limitation of the study.

In conclusion, patients with third-nerve palsies may regain acceptable eye alignments with this technique. Some patients may even regain limited adduction function after the procedure, whereas others may experience limitations in downgaze.

## Figures and Tables

**Table 1:** Demographic features, etiology, and clinical features of patients with total third-nerve palsy.

Patient number	Gender	Age at presentation (years)	Etiology	Type of 3rd-nerve palsy	Preoperative diplopia	Preoperative ptosis	Anisocoria	Follow-up (months)
1	M	33	Cranial trauma	Total	+	Moderate	+	11
2	M	36	Cavernous sinus hemangioma	Total	+	Moderate	+	40
3	M	14	Unknown etiology	Total	+	Mild	+	45
4	M	37	Hypophyseal adenoma	Total	−	Moderate	+	54
5	M	40	Carotid-cavernous fistula	Partial	+	−	−	37
6	M	45	Hypophyseal adenoma	Total	+	Mild	+	28

**Table 2:** Preoperative and postoperative deviations and surgeries performed.

Patient number	Eye involved	Preop. XT (PD)	Preop. hypotropia (PD)	Postop. deviation (PD)	Surgery performed (mm)
1	R	70	20	18 (XT) + 4 (Ho)	LR Rc 8, MR Rs 6, SOTr
2	L	70	15	15 (XT)	LR Rc 8, MR Rs 6, SOTr
3	R	65	12	8 (XT)	LR Rc 8, MR Rs 6, SOTr
4	L	70	10	8 (XT)	LR Rc 10, MR Rs 6, SOTr
5	R	50	20	4 (EP) + 4 (Ho)	LR Rc 7, MR Rs 5, SOTr
6	R	60	10	Orthotropia	LR Rc 8, MR Rs 6, SOTr

Preop. = preoperative. Postop. = postoperative. R = right. L = left. MR = medial rectus muscle. LR = lateral rectus muscle. SO = superior oblique muscle. Rc = recession. Rs = resection. Tr = transposition. Exotropia = XT. Esophoria = EP. Hypotropia = Ho. Prism diopter = PD.

Numbers indicate deviation in prism diopters and millimeters of surgery performed.

**Table 3:** Limitations of ocular movement at involved eyes.

Patient number	Limitations of ocular movement (graded between 0 and −4)			
	Abduction	Adduction	Depression	Elevation
1				
Preoperative	0	−4	−1	−3
Postoperative	−1	−2	−2	−2
2				
Preoperative	0	−4	−1	−2
Postoperative	0	−2	−2	−1
3				
Preoperative	0	−3	0	−2
Postoperative	0	−2	−2	−1
4				
Preoperative	0	−4	−1	−2
Postoperative	0	−2	−2	−1
5				
Preoperative	0	−2	−1	−3
Postoperative	−2	−1	−2	−2
6				
Preoperative	0	−3	−3	−3
Postoperative	−1	−1	−3	−3
